# Tackling Faradaic Imbalance in Redox Flow Batteries by the Use of a Solid Reducing Agent

**DOI:** 10.1021/acselectrochem.5c00520

**Published:** 2026-02-04

**Authors:** Gimena Marin-Tajadura, Ismael Suárez-Esteban, Ruben Rubio-Presa, Virginia Ruiz, Edgar Ventosa

**Affiliations:** † International Research Center in Critical Raw Materials-ICCRAM, 16725 University of Burgos, Pza. Misael Bañuelos s/n, E-09001 Burgos, Spain; ‡ Department of Chemistry, University of Burgos, Pza. Misael Bañuelos s/n, E-09001 Burgos, Spain

**Keywords:** state of health, faradaic imbalance, redox flow batteries, capacity fading, oxygen-induced parasitic reactions

## Abstract

Redox flow batteries (RFBs) represent a promising technology for large-scale energy storage. However, they suffer from capacity fading due to various factors, including desynchronization in the state of charge of the anolyte and catholyte, often caused by irreversible electrochemical side reactions. This study proposes a novel strategy to mitigate and reverse the effects of the faradaic imbalance by, for the first time to the best of our knowledge, introducing a solid reducing agent, LiFePO_4_ (LFP), in the catholyte compartment. The use of a heterogeneous reaction facilitates the removal of the reaction product, in contrast to homogeneous reducing agents. The strategy is implemented in a battery comprising K_4_Fe­(CN)_6_ as the catholyte and a viologen, 1,1′-bis­(3-sulfonatopropyl)-4,4′-bipyridinium (BSPV), as the anolyte in 1M KCl supporting electrolyte at neutral pH. The presence of trace oxygen in the anolyte leads to the accumulation of K_3_Fe­(CN)_6_ in the catholyte, resulting in a faradaic imbalance—used here as a case study. Introducing LFP pellets into the catholyte chemically reduces the accumulated K_3_Fe­(CN)_6_ back to K_4_Fe­(CN)_6_ via a spontaneous redox process, accompanied by the oxidation of LFP to FePO_4_, as confirmed by XRD analysis. Implementation of this method in a flow cell with a capacity-limiting catholyte results in a significant recovery of the lost capacity, which is attributed to the reduction of accumulated K_3_Fe­(CN)_6_ by the LFP pellets. This study presents a promising approach to addressing the faradaic imbalance in RFBs, potentially leading to improved performance and extended operational lifetime of these systems.

## Graphical abstract

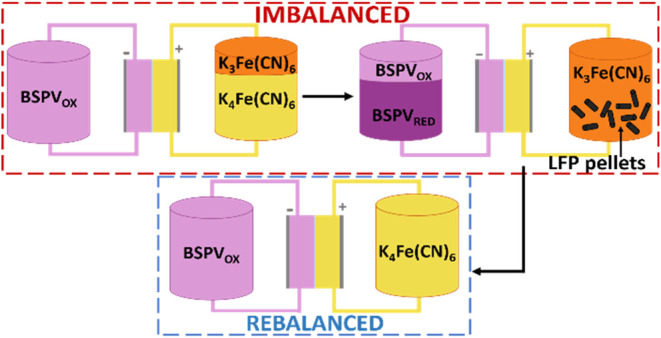

## Introduction

At the present time, society is striving to accelerate the energy transition by investing in renewable technologies to achieve the goals set at the European Green Deal.^1^ For the purpose of being able to depend only on renewable energy sources, there is a need to store the surplus energy of renewable energy sources to be used when required. With the aim of storing this energy, new battery technologies have been developed for large scale stationary energy storage, among which Redox Flow Batteries (RFBs) are a promising option.^2^ Vanadium flow batteries are the most commercially mature redox flow batteries. However, owing to the difficulty in accessing to vanadium reserves, new chemistries for flow batteries are being explored.^3^ Among these new technologies are aqueous organic redox flow batteries, which use aqueous electrolytes with organic molecules as energy-storage species. Although they are promising alternatives, they face some limitations, mainly a limited lifespan. Specifically, there are three primary sources of capacity fading in RFBs.^4^ The first one is the limitations of the flow cell, such as crossover, leakage, and membrane degradation. Researchers are actively addressing these cell limitations through various strategies. For example, the issue of crossover is approached by using bifunctional molecules as catholytes and anolytes simultaneously or by having both species, the anolyte and catholyte, on both sides, as in the case of symmetric batteries.^5,6^ The second source of capacity fading is the chemical/electrochemical instability of the molecules that store the energy, which can be addressed by molecular engineering.^7–9^ The last source is the state of charge imbalance between both compartments, which is due to the occurrence of irreversible electrochemical side reactions in both tanks, the catholyte and anolyte. The main side reactions that occur in RFBs are hydrogen evolution reaction (HER), oxygen evolution reaction (OER), and oxygen-induced parasitic reactions. The latter leads to self-discharge (spontaneous reoxidation) of the anolyte due to traces of oxygen, which occurs to some extent when the cell is operated outside an Ar-filled glove box. At an industrial scale, it is challenging to completely prevent this issue over many years of operation, considering its accumulative nature. Therefore, strategies to address this challenge must be developed. The various approaches can be categorized in electrochemical strategies and chemical strategies.^10^ In the former, the accumulated charged catholyte species are electrochemically reduced using auxiliary electrochemical systems to restore the charge balance, which has been shown to be effective for AVRFB^11^ and AORFB.^4,12^ The environmental friendliness (only oxygen is generated as waste) is the main advantage of electrochemical strategies, while the increased complexity of the systems is one of the main drawbacks. In the latter, a chemical reducing reactant is added to the electrolyte, so that the accumulated charged catholyte species are chemically reduced through a homogeneous redox reaction. For example, in all-vanadium flow batteries, hydrogen evolution and the ingest of oxygen into the anolyte compartment lead to the accumulation of the charged catholyte species V^5+^ (VO_2_^+^). The addition of organic reducing agents, such as oxalic acid,^13–15^ in the catholyte was shown to reverse the effect of the faradaic imbalance.^16^ Importantly, the reducing agent is oxidized to CO_2_, leaving no interfering residues in the catholyte, as called the waste-free method. In this specific case, the acidity of the medium and the high redox potential of the catholyte (+1.0 V vs SHE) allow the use of such homogenous reducing agents. On the other hand, to the best of our knowledge, heterogeneous (solid) reducing agents have not been explored to reverse the effect of faradaic imbalance.

The simplicity is the main advantage of chemical approaches, while its implementation is not universal since the pH and redox potential of the catholyte determine the choice of the reducing agent. For example, the reducing agent proposed in the literature (organic acid) cannot be implemented in neutral and alkaline redox flow battery chemistries since the redox potentials of the catholyte in neutral and alkaline media are lower than that of V^4+^/V^5+^ species (thermodynamic limitations due to stability window of the electrolyte), and the pH of the media is influenced when adding the reducing agent (organic acids).

In this context, we propose a new strategy to reverse the effects of the faradaic imbalance by, for the first time to the best of our knowledge, introducing a solid reducing agent into the catholyte to rebalance the state of charge between both compartments. The use of a heterogeneous reducing agent facilitates its removal or even its confinement in the tank, thanks to the higher energy density of solid reducing agents than a liquid alternative.

## Experimental Section

### Materials

All common reagents and solvents were analytical grade and used as received without further purification. Potassium ferrocyanide trihydrate, potassium ferricyanide, poly­(vinylidene fluoride), and *N*-methyl-2-pyrrolidone were purchased from Thermoscientific. Potassium chloride was purchased from Aldrich and Ketjen Black from Nanografi. Standard clear resin for the 3D printer was purchased from Anycubic. Solutions were prepared by using deionized water.

### Preparation of LFP Pellets

LFP pellets were prepared by extruding a mixture of LFP, KB, and PVDF in NMP with a 80:10:10 (LFP/KB/PVDF) composition from a 20 mL syringe. Obtained filaments with 2 mm diameter were cut into 7 mm length pieces and dried in an oven at 60 °C for 24 h. FP pellets were prepared by oxidizing LFP pellets and soaking them in a 0.2M K_3_Fe­(CN)_6_ oversized solution for 3 days.

### Flow Cells

Filter-pressed flow cells using Nafion 212 (no previous soaking) as the ion-selective membrane and graphite felt (Sigracell GFD 2.5, SGL Carbon) as electrodes were used for the flow experiments. The graphite felt is compressed by approximately 20% during the assembly of the cell. Graphite felt was electrochemically activated in the assembled cell by applying a constant current of 7 mA for 2 h per side with 50 mL of solutions of 1 M KOH in each side of the cell. The projected area of the cell was 10 cm^2^ and the flow rate was fixed at 40 mL min^–1^. General conditions for the flow cell were as follows: anolyte 11 mL of 0.4M BSPV in 1.0 M KCl and catholyte 15 mL of K_4_Fe­(CN)_6_ in 1.0 M KCl.

### Electrochemical Characterization

Electrochemical measurements were performed using a Neware battery testing system (BTS), Neware BTS model -4008Tn-5 V6A–S1-F. The flow cells were galvanostatically cycled with 20 mA cm^–2^ with voltage limits of 1.1 V during the charge and 0.5 V during the discharge.

### X-ray Diffraction Measurements

XRD measurements were conducted using a Bruker D8 Discover diffractometer with KFL Cu radiation in the range of 10-60 °(2θ) and a step of 0.02 °.

### UV-Vis Measurements

UV-Vis spectra were measured using a CARY 50 Conc of Varian.

## Results and Discussion

The main objective of this work is to demonstrate the feasibility of using solid reducing agents through heterogeneous reactions to reverse the effects of the faradaic imbalance, which poses intrinsic advantages in comparison with homogeneous reducing agents, such as easy recovery of the product or even confinement in the tank (the higher energy density of solid materials). As a proof-of-concept, a RFB with commonly used pair of redox electrolytes was studied, K_4_Fe­(CN)_6_ as catholyte and a viologen derivative, 1,1′-Bis­(3-sulfonatopropyl)-4,4′-bipyridinium (BSPV), as anolyte in 1M KCl neutral pH supporting electrolyte. In this battery, the parasitic reaction due to the traces of oxygen in the anolyte is used as a case study, preventing the anolyte from reaching the full state of charge (Figure 1). Note that oxygen is absent in anolyte during charge from the proposed reactions with rebalancing to simplify the scheme. This in turn limits the extent to which the catholyte can be later discharged, which leads to the accumulation of charged species in the catholyte (K_3_Fe­(CN)_6_) at the end of each charge/discharge cycle. This imbalance in the state of charges between the catholyte and the anolyte is known as faradaic imbalance. Our strategy consists of introducing a solid reducing agent in the catholyte to chemically reduce the accumulated K_3_Fe­(CN)_6_ to its discharged form, K_4_Fe­(CN)_6_, thus counteracting the unbalancing effect originated from the presence of traces of oxygen in the anolyte. Specifically, the “rebalancing solid” used was LiFePO_4_ (LFP) because its redox potential is lower than that of K_3_Fe­(CN)_6_, which drives the spontaneous redox process between LFP and K_3_Fe­(CN)_6_ whereby K_3_Fe­(CN)_6_ is reduced, while LFP is oxidized.

**Figure 1:**
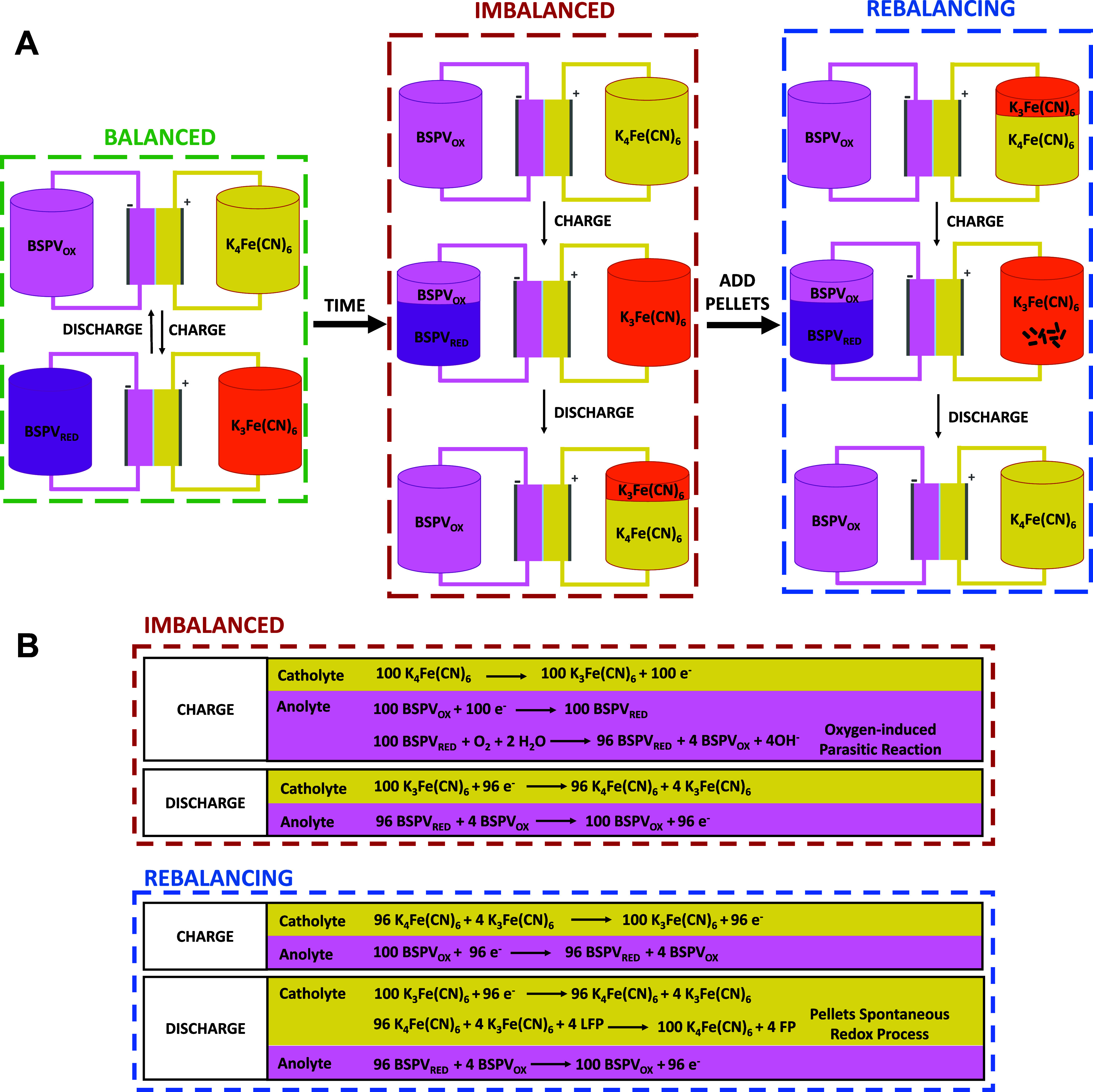
(A) Schematic representation of the oxidation state of the tanks in a BSPV/K_4_Fe­(CN)_6_ AORFB at the end of charge and discharge. The battery is initially balanced at the beginning of the cycling (green dashed line), but over time it becomes imbalanced due to the occurrence of oxygen-induced parasitic reactions in the negative tank, which lead to an imbalance between the state of charge of both tanks (red dashed line). The last scheme (blue dashed line) represents the rebalancing process when LFP pellets are added to the catholyte compartment. (B) Reactions occurring during charge and discharge in an imbalanced and rebalanced BSPV/K_4_Fe­(CN)_6_ flow battery. Numbers in the reactions represent with arbitrary units a hypothetical situation where oxygen enters the anolyte compartment (dashed red line, imbalanced) and the rebalancing reaction at the catholyte when LFP pellets are introduced (dashed blue line, rebalanced).

Before implementing the strategy, we first demonstrated that the capacity loss is caused by the faradaic imbalance, leading to accumulation of K_3_Fe­(CN)_6_ in the catholyte throughout the cycles. To do so, a battery using the catholyte (15 mL of 0.2 M K_4_Fe­(CN)_6_ in 1 M KCl) and the anolyte (11 mL of 0.4 M BSPV in 1 M KCl) as capacity-limiting side (CLS) and non-capacity limiting side (NCLS), respectively, was galvanostatically charged and discharged. A progressive capacity decay was noted during the first 48 cycles (Figure 2). Once the battery had lost nearly 40% of its initial capacity, the catholyte was replaced by a fresh solution (15 mL of 0.2 M K_4_Fe­(CN)_6_), which enabled the recovery of most of the lost capacity. Note that the accelerated ingest of oxygen also affects the cycle stability of the anolyte due to the resulting increase in pH (oxygen reduction). Thus, no full recovery is attributed to the degradation of the anolyte via dealkylation through a nucleophilic attack of hydroxide anions.^17^ In order to further investigate the origin of capacity decay (whether due to active material degradation or the accumulation of K_3_Fe­(CN)_6_), the cycled catholyte (removed after 48 cycles) was transferred to a new cell (Figure S3). In this step, the aged catholyte acted as CLS while the NCLS contained 100 mL of a 0.1 M K_3_Fe­(CN)_6_ and 0.1 M K_4_Fe­(CN)_6_ mixture. The capacity was measured during the first oxidation of the aged catholyte in the new cell (Figure S3) and matched the value obtained in the last cycle before its removal (45.4 mAh, Figure 2). Importantly, the capacity during the subsequent reduction step was identical to that of the first cycle in the original battery (74.3 mAh). This recovery of capacity confirms that the catholyte did not undergo irreversible degradation. Instead, the observed capacity fading in Figure 2 is attributed to a faradaic imbalance and a reversible accumulation of charge in the catholyte. Additionally, the results in Figure S3 also support that the no full recovery in Figure 2 after exchanging the catholyte are due to partial degradation of the anolyte.

**Figure 2:**
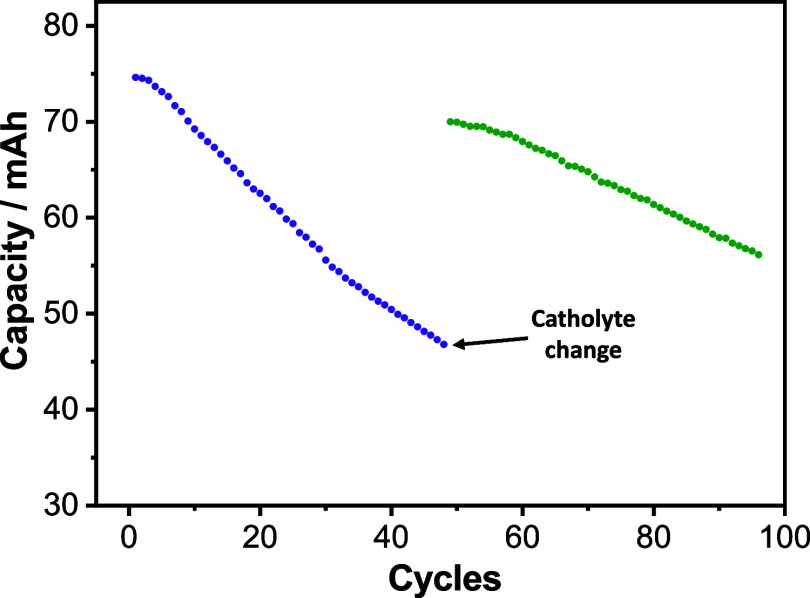
Evolution of discharge capacity with the number of cycles of a flow battery with a catholyte of 15 mL of 0.2 M K_4_Fe­(CN)_6_ and an anolyte of 11 mL of 0.4 M BSPV in 1 M KCl, cycled with a constant current of 20 mA/cm^2^ and cut-offs of 0.5 and 1.1 V. The purple dots correspond to the battery cycled for 48 cycles, at which time the aged catholyte was replaced by the same volume of a fresh solution with the same composition, 15 mL of 0.2 M K_4_Fe­(CN)_6_ in 1 M KCl, whose capacity is represented by the green dots.

Therefore, the faradaic imbalance is identified as the primary cause of capacity fading under the specific conditions of our experiment. It is worth noting that although the rate of oxygen ingress can be reduced, the suboptimal performance of the system actually played in our favor in this case study by accelerating the manifestation of the imbalance. To evaluate the feasibility of using LFP as a solid reducing agent to reverse the effects of a faradaic imbalance, two identical electrochemical cells were assembled. Each cell contained 15 mL 0.2 M K_4_Fe­(CN)_6_ as the CLS and 100 mL of 0.1 M K_3_Fe­(CN)_6_ / 0.1 M K_4_Fe­(CN)_6_ mixture as the NCLS. The CLS compartment underwent two cycles of oxidation and reduction at a constant current density of 20 mA/cm^2^, with voltage cut-offs set at 0.35 V and −0.35 V, respectively. Capacity values for the oxidation and reduction steps of the two cells are summarized in Table S1. Note that an external reservoir using an SLA 3-D printer was fabricated to facilitate the addition and reaction of the LFP material. A coulombic inefficiency was consistently observed in the first cycle for all experiments when this 3-D printed reservoir was used (the two experiments in Table S1 and the one experiment in Table S2), while it does not occur when a conventional tank (e.g., polypropylene) is used (Figure S4). Thus, we attribute this to the use of the 3-D printed reservoir. At this moment, the exact source is under investigation since it cannot be unambiguously attributed to a reducing nature of the polymer (causing part of the generated ferricyanide to be reduced back to ferrocyanide, extending the first oxidation cycle) or other sources (e.g., flow distribution). In the comparative tests with the two cells, an additional oxidation step was conducted to fully charge the CLS to 0.2 M K_3_Fe­(CN)_6_, followed by an open circuit potential (OCP) of 2 h (Table S1). At the beginning of the OCP step, 0.50 g of LFP pellets (80 LFP:10 KB:10 PVDF) were introduced into the CLS compartment of only one of the two cells. After the OCP step, the cells were subjected to another oxidation step. In this step, the cell containing the LFP pellets delivered a capacity of 41.6 mAh, whereas the reference cell (without pellets) delivered a negligible capacity of 0.1 mAh (Table S1). Note that 0.1 mAh accounts for less than 0.2 % of the electrolyte capacity and it is attributed to the homogenization of the electrolyte plus capacitive contribution after the resting period. After a second OCP period of 2 h, another oxidation step was applied to the CLS compartment of both cells, and again only the cell containing pellets delivered a significant capacity of 18.5 mAh as opposed to the null capacity provided by pellet-free reference cell. That is, the cell containing the LFP pellets demonstrated a total additional capacity of 60.1 mAh, corresponding to the oxidation of 0.50 g of pellets. Based on a theoretical specific capacity of 150 mAh g^–1^ for LFP,^18^ the calculated theoretical capacity of the added pellets (which contained 80% LFP) was 60 mAh, in close agreement with the experimentally measured value. These LFP pellets were oxidized to FP with the concomitant reduction of K_3_Fe­(CN)_6_ through a spontaneous redox process. To confirm the oxidation of the LFP pellets, XRD measurements were conducted on both the pristine LFP pellets and those removed from the cell, where they were supposed to be oxidized by reaction with K_3_Fe­(CN)_6_. The XRD pattern of the pristine LFP pellets displayed (2 0 0) and (2 1 0) diffraction peaks at 17.2° and 22.7°, respectively, attributed to the LiFePO_4_ phase, while the oxidized pellets exhibited the characteristic (2 0 0) and (2 1 0) diffraction peaks of the FePO_4_ phase at 18.0° and 23.7°, respectively (Figure S5). These findings confirmed the oxidation of LFP pellets in contact with the K_3_Fe­(CN)_6_ solution during the OCP step.

To explore the efficiency of the rebalancing process (in terms of the LFP utilization rate), a dose-response study was conducted. The electrolyte in the CLS was intentionally set to 50 % state of charge (emulating a catholyte imbalance of 50 %). Four experiments were conducted in which the amount of LFP pellets (80 LFP: 10 KB: 10 PVDF) added to the tank was changed, namely 0.25 g, 0.5, 0.75, and 1 g (Figure S6). The increase in charge storage after the addition of the LFP pellets was compared to the theoretical charge capacity of the added LFP (150 mAh g^–1^ × *X* g of LFP, where *X* is the amount of LFP). For 0.25, 0.5, and 0.75 g, the utilization rate of the charge capacity of the LFP was almost 100 % (Figure S6B). At 0.75 g, the full electrolyte capacity was recovered, so that the addition of more LFP did not lead to any further improvement, resulting in a drop in the utilization rate since more LFP than needed is added. Thus, the amount of the LFP to be added should be estimated beforehand, considering the charge capacity to be recovered and the charge capacity of the added LFP and knowing that the utilization rate of the LFP is very close to 100 %. Furthermore, the experiment corresponding to the addition of 0.75 g was chosen to assess the longer-term performance. While the rebalancing capacity of the LFP ceases when the solid material is oxidized (acting as reducing agent), the impact of having FP pellets in the tank in the longer term was evaluated. Figure S7 shows that the electrolyte capacity increased from *ca*. 82 (imbalanced) to 172 mAh (rebalanced) after the addition of 0.75 g of LFP, and this value remained very stable (capacity fading of 0.007 % cycle^–1^) for the 200 cycles tested (over a week), indicating that the presence of FP in the tank does not influence negatively the cycling stability.

Once the reducing capability of LFP pellets for K_3_Fe­(CN)_6_ electrolyte and its utilization rate was demonstrated, it was important to ensure that the “rebalancing material” can selectively and irreversibly reduce the charged catholyte, without promoting the undesired reverse reactions, such as the oxidation of the catholyte. Indeed, the first evidence is found in Table S1. When LFP is added, the oxidation step accumulated 137.9 mAh (77.8 + 41.6 + 18.5 mAh), while the reduction step delivered only 78.6 mAh, suggesting that the charge transfer between catholyte and LFP occurs only in one direction. In order to further demonstrate that FP is not capable of oxidizing K_4_Fe­(CN)_6_, an additional electrochemical flow cell was assembled and tested. As in previous experiments, the flow cell consisted of 15 mL of 0.2 M K_4_Fe­(CN)_6_ as the CLS and 100 mL of 0.1 M K_3_Fe­(CN)_6_ and 0.1 M K_4_Fe­(CN)_6_ as the NCLS. The CLS compartment underwent two cycles of oxidation and reduction at a constant current density of 20 mA/cm^2^, with voltage cut-offs set at 0.35 V and −0.35 V, respectively. Capacity values for the oxidation and reduction steps are summarized in Table S2. In this case, the OCP step was initiated after the reduction step to ensure that the catholyte was in its fully reduced form, 0.2 M of K_4_Fe­(CN)_6_. At the beginning of this OCP step, FP pellets (oxidized LFP pellets) were introduced into the CLS compartment. After this OCP period of 2 h, the CLS was subjected to another reduction step to evaluate whether the FP pellets were able to oxidize the K_4_Fe­(CN)_6_. No capacity was measured during this subsequent reduction (Table S2). The OCP and reduction steps were repeated, yet no capacity was obtained in any of these reductions, indicating that the redox species in the CLS remain in its fully reduced form, K_4_Fe­(CN)_6_, after addition of FP pellets. This finding confirmed the irreversible oxidation of LFP in this system. For further confirmation, XRD measurements were conducted to the FP pellets before and after being extracted from the CLS of the electrochemical cell. Both pellets exhibited exactly the same diffraction peaks, (2 0 0) and (2 1 0), corresponding to the FePO_4_ phase at 18.0° and 23.7° (Figure S8), which provide additional evidence of the irreversible oxidation state of FP.

Once it was confirmed that the capacity loss was due to the faradaic imbalance and that LFP has the ability to chemically reduce K_3_Fe­(CN)_6_ in symmetric, compositionally unbalanced K_4_Fe­(CN)_6_/K_3_Fe­(CN)_6_ flow cells, we implemented this new rebalancing strategy to mitigate and reverse the capacity loss induced by the oxygen-induced self-discharge of the anolyte in a flow battery. The system used to validate this new method consisted of 15 mL of 0.2 M K_4_Fe­(CN)_6_ as the catholyte and 11 mL of 0.4 M BSPV as the anolyte in 1 M KCl. This battery was cycled at a constant current of 20 mA/cm^2^ with cut-offs of 0.5 and 1.1 V for the discharge and charge, respectively. The battery was cycled until it lost 30 % of its initial capacity (cycle 72, Figure 3A) and, at this point, cycling was paused to take a 5 μL aliquot of the catholyte and to add 0.26 g of LFP pellets to the catholyte. The aliquot was diluted in 2 mL of 1 M KCl and the absorbance of the resulting solution was measured by UV-Vis absorption spectroscopy to confirm that the capacity loss was due to the accumulation of K_3_Fe­(CN)_6_ in the catholyte during cycling. Indeed, K_3_Fe­(CN)_6_ was detected in the catholyte at the end of the discharge step of the 72nd cycle estimating a concentration of 57 mM (Section S7), and confirming charge accumulation in the catholyte (faradaic imbalance). As the initial concentration of K_4_Fe­(CN)_6_ was 0.2 M, the 30 % capacity loss noted by the 72nd cycle amounts to a concentration of accumulated K_3_Fe­(CN)_6_ of 60 mM, which is very close to the value determined by UV-Vis absorption spectroscopy. After the pause that was applied to add LFP pellets to the catholyte, the battery continued cycling. During the first cycles after the pause, the capacity increased until almost reaching the initial value (Figure 3A). This capacity recovery is attributed to the reduction of accumulated K_3_Fe­(CN)_6_ by the LFP pellets added to the catholyte. When the LFP in the pellets was fully oxidized, the capacity started to drop again as the oxygen entering the anolyte compartment keeps unbalancing the battery. The added pellets were characterized by XRD before and after being added to the battery catholyte. Note that the purpose of using XRD in these experiments was to confirm whether the solid material is in its oxidized or reduced form. Thus, a quantitative analysis was not conducted since detecting the presence of impurities is not relevant in this case (the oxidation of LFP is irreversible). As shown in Figure 3C, the LFP pellets added to the catholyte were in its reduced form with the characteristic (2 0 0) and (2 1 0) diffraction peaks of LiFePO_4_ at 17.2° and 22.7°, respectively. Meanwhile, the pellets extracted from the battery at the end of the cycling (Cycle 165) were in its oxidized form, exhibiting the characteristic peaks of the FePO_4_ phase and (2 0 0) and (2 1 0) diffraction peaks at 18.0° and 23.7°, respectively. Note that other characteristic peaks of the FePO_4_ phase are highlighted with asterisks in the full-scale pattern.

**Figure 3:**
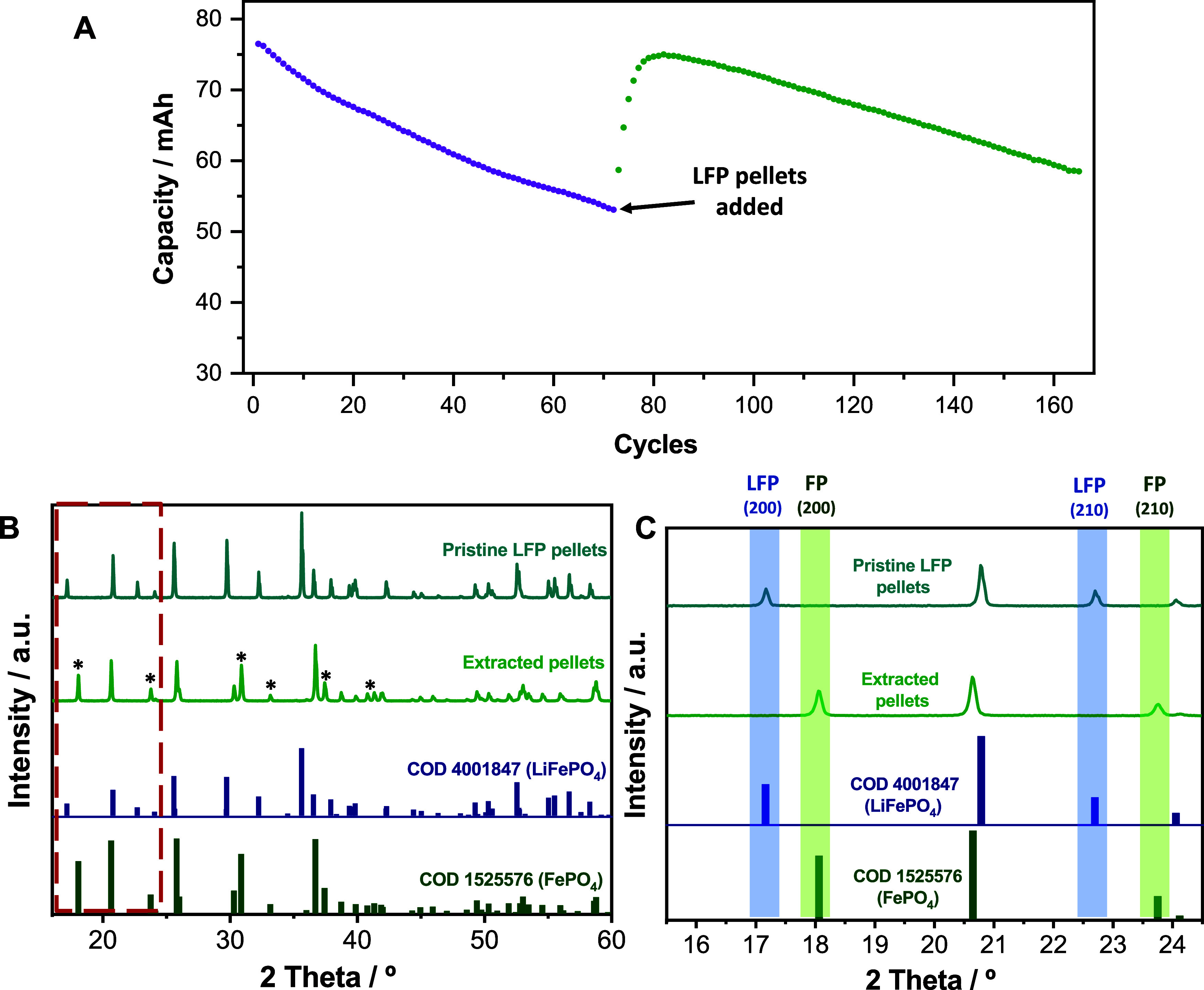
(A) Evolution of capacity during cycling of a K_4_Fe­(CN)_6_/BSPV flow battery with 15 mL of 0.2 M K_4_Fe­(CN)_6_ catholyte and 11 mL of 0.4 M BSPV in 1 M KCl anolyte at a constant current density of 20 mA/cm^2^ and cut-offs of 0.5 and 1.1 V. The purple dots correspond to the battery before adding the pellets and the green dots for the battery after adding the pellets. Full (B) and zoomed (C) XRD patterns of pristine LFP pellets and extracted pellets at the end of the battery cycling. Characteristic peaks of the FePO_4_ phase are highlighted with asterisks.

Bearing in mind the fundamental nature of this work, there are two important aspects considering the practicality of the proposed approach; its implementation and its cost effectiveness. Regarding the implementation of this approach, there are various options. For instance, a packed bed reactor with valves that is eventually flooded with the electrolyte is a possibility. Simple addition of solid electrodes when the battery is fully charged waiting until the reaction has ended to retrieve them is also possible, depending on the kinetics. But even simpler is the addition of pellets, leaving them at the bottom of the tank. This is possible due to the much higher volumetric capacity of LFP compared to the electrolyte. Namely, the volumetric capacity of LFP is around 500 Ah L^–1^, while a catholyte containing 1 M of active species would have 26 Ah L^–1^, which is 20 times lower than that of LFP. This means that recovering 50 % of the capacity (13 Ah L^–1^) would require an increase in the tank volume of 2.6 %. Considering the porosity of the pellets, this value could increase up to 5 %, which is still reasonable. In that case, the accumulation of Li^+^ would not be a limitation since half of it would diffuse to the anolyte so that the concentration of Li^+^ would be 25 % of that of K^+^ (ferrocyanide is more soluble in Li^+^ electrolyte).^19^ Regarding the cost effectiveness, the choice will depend on the chemistry. In this particular case, both catholyte and solid reducing agent are based on Fe. The cost effectiveness of the rebalancing versus replacing the entire electrolyte approach would depend on the state of health at which the battery undergoes reconditioning. That is, if only 20 % is to be recovered, addition of LFP would likely be cheaper than replacing the entire catholyte. However, if 80 % of the capacity is to be recovered, replacing the electrolyte might be even cheaper. However, the rebalancing approach has a clear technoeconomic advantage; small capacity losses can be easily recovered. By doing this, the energy storage capacity of the battery can be continuously maintained between 95 and 100% of the original capacity. Replacing the electrolyte requires that the capacity drops below a certain value, e.g., 70 % to be cost efficient, which implies that the battery operates below its nominal value for several years. The latter scenario is not desired by the operator of the battery.

## Conclusions

In summary, we proposed a novel strategy to mitigate and reverse the effects of the faradaic imbalance caused by accumulation of charged catholytes due to the occurrence of parasitic side reactions in the anolyte of aqueous redox flow batteries (RFBs). This parasitic reaction prevents the anolyte from reaching the full state of charge, which in turn limits the achievable state of discharge of the catholyte in the following cycle. This leads to a progressive accumulation of charged species in the catholyte during battery operation. A straightforward strategy to counteract this charge imbalance between both compartments has been demonstrated in a K_4_Fe­(CN)_6_/BSPV flow battery, which consists in adding -for the first time to the best of our knowledge- a solid reducing agent, LiFePO_4_ (LFP), in the catholyte compartment to reduce the accumulated K_3_Fe­(CN)_6_ back to its discharged form, K_4_Fe­(CN)_6_. The efficiency of the method has been demonstrated in both symmetric cell and full battery configuration, allowing us to recover most of the lost capacity. The rebalance mechanism, selective and irreversible chemical reduction of accumulated K_3_Fe­(CN)_6_ to K_4_Fe­(CN)_6_ by LFP, has been confirmed by electrochemical and XRD measurements. The simplicity, efficiency, and scalability of the rebalancing strategy hold great potential for enhancing the performance and longevity of RFBs in the future.

## Supplementary Material



## Notes

Additional experimental details, materials, and methods (PDF)

## References

[1] Olczyk M., Kuc-Czarnecka M. (2025). European Green Deal Index: A New Composite Tool for
Monitoring European Union’s Green Deal Strategy. J. Clean. Prod..

[2] Sánchez-Díez E., Ventosa E., Guarnieri M., Trovò A., Flox C., Marcilla R., Soavi F., Mazur P., Aranzabe E., Ferret R. (2021). Redox Flow Batteries:
Status and
Perspective towards Sustainable Stationary Energy Storage. J. Power Sources.

[3] Lee W., Park G., Shin M., Emmel D., Schröder D., Kwon Y. (2025). Challenges and Advances
in Redox Flow Batteries Utilizing Sustainable
and Cost-Effective Non-Vanadium Active Materials. J. Mater. Chem. A.

[4] Páez T., Martínez-Cuezva A., Marcilla R., Palma J., Ventosa E. (2021). Mitigating Capacity Fading in Aqueous Organic Redox
Flow Batteries through a Simple Electrochemical Charge Balancing Protocol. J. Power Sources.

[5] Luo J., Hu B., Debruler C., Bi Y., Zhao Y., Yuan B., Hu M., Wu W., Liu T. L. (2019). Unprecedented Capacity and Stability
of Ammonium Ferrocyanide Catholyte in PH Neutral Aqueous Redox Flow
Batteries. Joule.

[6] Potash R. A., McKone J. R., Conte S., Abruña H. D. (2016). On the
Benefits of a Symmetric Redox Flow Battery. J. Electrochem. Soc..

[7] Pan M., Shao M., Jin Z. (2023). Development
of Organic Redox-Active
Materials in Aqueous Flow Batteries: Current Strategies and Future
Perspectives. SmartMat.

[8] Mansha M., Ayub A., Khan I. A., Ali S., Alzahrani A. S., Khan M., Arshad M., Rauf A., Akram Khan S. (2024). Recent Development
of Electrolytes for Aqueous Organic Redox Flow Batteries (Aorfbs):
Current Status, Challenges, and Prospects. Chem.
Rec..

[9] Wedege K., Dražević E., Konya D., Bentien A. (2016). Organic Redox
Species in Aqueous Flow Batteries: Redox Potentials, Chemical Stability
and Solubility. Sci. Rep..

[10] Nolte O., Volodin I. A., Stolze C., Hager M. D., Schubert U. S. (2021). Trust Is
Good, Control Is Better: A Review on Monitoring and Characterization
Techniques for Flow Battery Electrolytes. Mater.
Horiz..

[11] Poli N., Schäffer M., Trovò A., Noack J., Guarnieri M., Fischer P. (2021). Novel Electrolyte Rebalancing Method for Vanadium Redox
Flow Batteries. Chem. Eng. J..

[12] Lubian L., Rubio-Presa R., Ruiz V., Colina A., Ventosa E. (2025). Raman Spectroelectrochemistry
for Operando Characterization of Redox Flow Batteries. J. Power Sources.

[13] Li W., Zaffou R., Sholvin C. C., Perry M. L., She Y. (2013). Vanadium Redox-Flow-Battery
Electrolyte Preparation with Reducing Agents. ECS Trans..

[14] Skyllas-Kazacos, M. ; Michael Kazacos, M. ; Mcdermott, R. J. C. Vanadium compound dissolution processes. WO Patent WO1989005363A1 1988.

[15] Keshavarz, M. ; Zu, Ge. Production of vanadium electrolyte for a vanadium flow cell. U.S. Patent US20150050570A1 2014.

[16] Chang, O. K. ; Pham, A. Q. Rebalancing Electrolytes in Redox Flow Battery Systems. U.S. Patent US8916281B2 2014.

[17] Rubio-Presa R., Lubián L., Borlaf M., Ventosa E., Sanz R. (2023). Addressing
Practical Use of Viologen-Derivatives in Redox Flow Batteries through
Molecular Engineering. ACS Mater. Lett..

[18] Ramasubramanian B., Sundarrajan S., Chellappan V., Reddy M. V., Ramakrishna S., Zaghib K. (2022). Recent Development in Carbon-LiFePO4 Cathodes for Lithium-Ion
Batteries: A Mini Review †. Batteries.

[19] Li X., Yao Y., Jia X., Liu C., Jian J., Guo B., Cui K., Lu S., Li Y., Qin W., Wang Q., Wu X. (2023). Lithium Ferrocyanide
Catholyte for High-Capacity Aqueous Redox Flow
Batteries. Angew. Chem., Int. Ed..

