# Supplementary material for: Tackling Faradaic Imbalance in Redox Flow Batteries by the Use of a Solid Reducing Agent
Source: ACS Electrochem. 2026 Feb 4;2(3):745–51. doi: 10.1021/acselectrochem.5c00520 (PMC12969641; doi:10.1021/acselectrochem.5c00520)
Supplement: Supplementary file 1 [file ec5c00520_si_001.pdf]

Supporting Information

Tackling Faradaic Imbalance in Redox Flow Batteries by the Use of a Solid Reducing Agent

Gimena Marin-Tajadura, Ismael Suárez-Esteban, Ruben Rubio-Presa, Virginia Ruiz, Edgar Ventosa**

G. Marin-Tajadura, I. Suárez-Esteban, R. Rubio-Presa, V. Ruiz, E. Ventosa

International Research Center in Critical Raw Materials-ICCRAM, University of Burgos, Pza. Misael Bañuelos s/n, E-09001, Burgos, Spain.

Department of Chemistry, University of Burgos, Pza. Misael Bañuelos s/n, E-09001-Burgos, Spain.

E-mail: vrfernandez@ubu.es and eventosa@ubu.es

Table of contents

Section S1. Synthesis and characterization of 1,1′-Bis(3-sulfonatopropyl)-4,4′-bipyridinium (BSPV)....2

Section S2. Confirmation of $K_3Fe(CN)_6$ accumulation in the catholyte during cycling...4

Section S3. Efficiency of the first cycle of a cell with a conventional tank....5

Section S4. Reduction capability of LFP....6

Section S5. Efficiency of the rebalancing process with different amounts of LFP and long-term performance....7

Section S6. Irreversible oxidation of LFP in $K_4Fe(CN)_6$ catholyte....9

Section S7. UV-Vis calibration for determination of $K_3Fe(CN)_6$ concentration....11

References 12

Section S1. Synthesis and characterization of 1,1′-Bis(3-sulfonatopropyl)-4,4′-bipyridinium (BSPV)

Synthesis

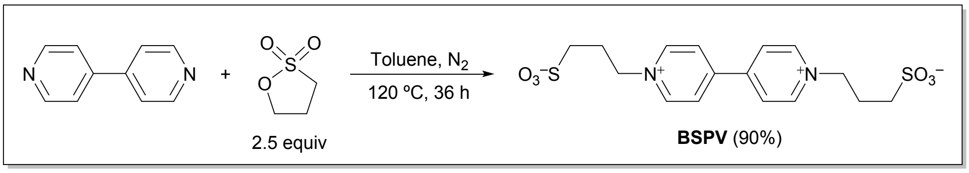

BSPV was prepared according to a reported method.[1]

In a round bottom flask (250 mL), 1,3-propanesultone (9.8 g, 80 mmol) was dissolved in anhydrous toluene (60 mL) under an inert nitrogen atmosphere and the obtained solution was heated at 110 °C. Then, a solution of 4,4′-bipyridine (5 g, 32 mmol) in anhydrous toluene (40 mL) was added slowly with stirring. The reaction mixture was heated at reflux (oil bath, 120 °C) with vigorous stirring for 36 h. Upon cooling to room temperature, the resulting white precipitate was filtered off, washed several times with hot MeOH and dried under vacuum.

The spectral data of the compound are in good agreement with the reported data.

1,1′-Bis(3-sulfonatopropyl)-4,4′-bipyridinium: Yielded (11.22 g, 90%) as a white solid.

Characterization

^{1}H NMR (300 MHz, D_2O): δ = 9.18 (d, *J* = 6.9 Hz, 4H, Ar*H*), 8.59 (d, *J* = 6.6 Hz, 4H, Ar*H*), 4.92 (t, *J* = 7.4 Hz, 4H, CH_2), 3.06 (t, *J* = 7.2 Hz, 4H, CH_2), 2.56 (ap, *J* = 7.3 Hz, 4H, CH_2).

^{13}C NMR (75.4 MHz, D_2O): δ = 150.4 (2 × C), 145.7 (4 × CH), 127.2 (4 × CH), 60.2 (2 × CH_2), 47.0 (2 × CH_2), 26.2 (2 × CH_2).

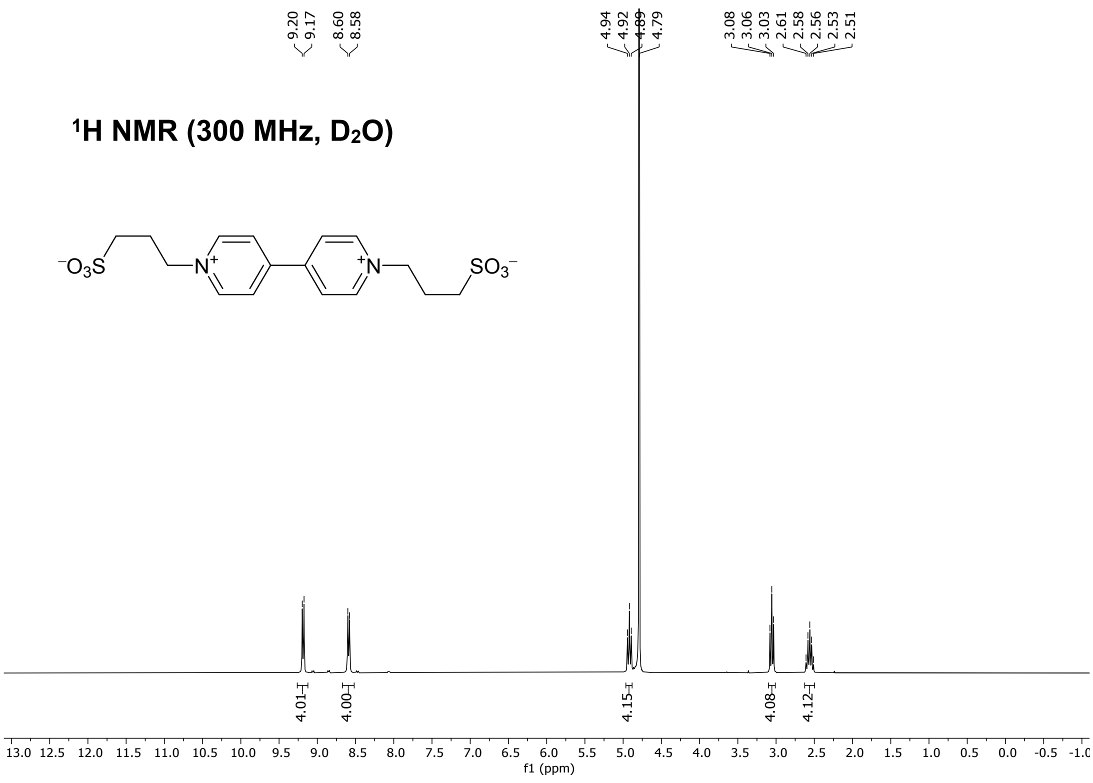

Figure S1. ^{1}H-NMR spectrum of BSPV.

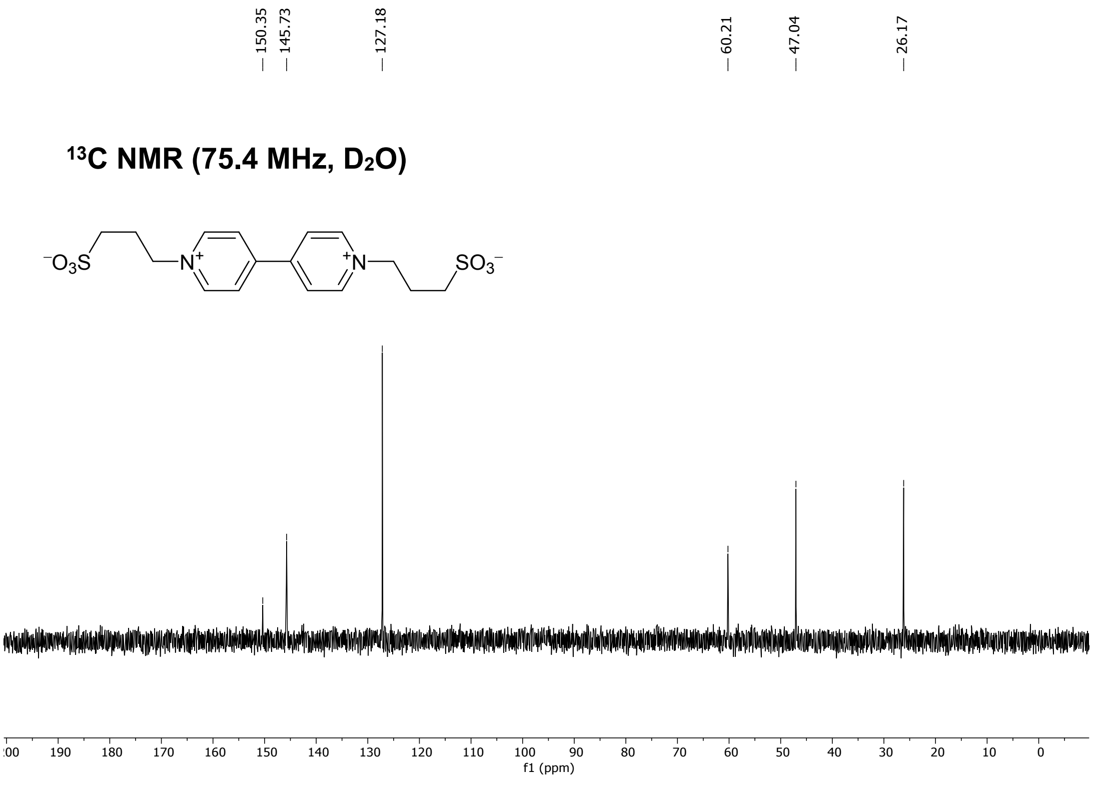

Figure S2. ^{13}C-NMR spectrum of BSPV.

Section S2. Confirmation of $K_3Fe(CN)_6$ accumulation in the catholyte during cycling

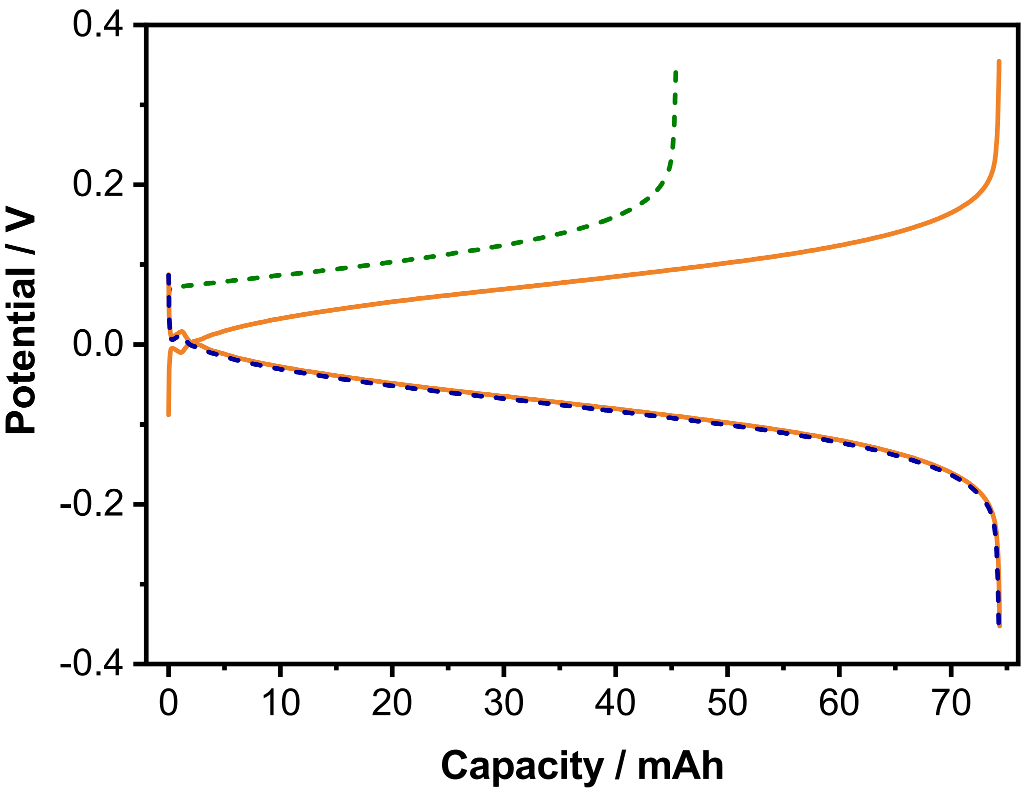

Figure S3. Voltage profiles for the oxidation and reduction of the aged catholyte from a previous battery (Figure 2) as the CLS using an oversized counter electrode comprising 100 mL of 0.1 M $K_3Fe(CN)_6$ and 0.1 M $K_4Fe(CN)_6$. Green dashed line corresponds to the first oxidation of the CLS electrolyte (aged catholyte), blue dashed line to the first reduction and the orange lines correspond to the second oxidation and reduction.

Section S3. Efficiency of the first cycle of a cell with a conventional tank

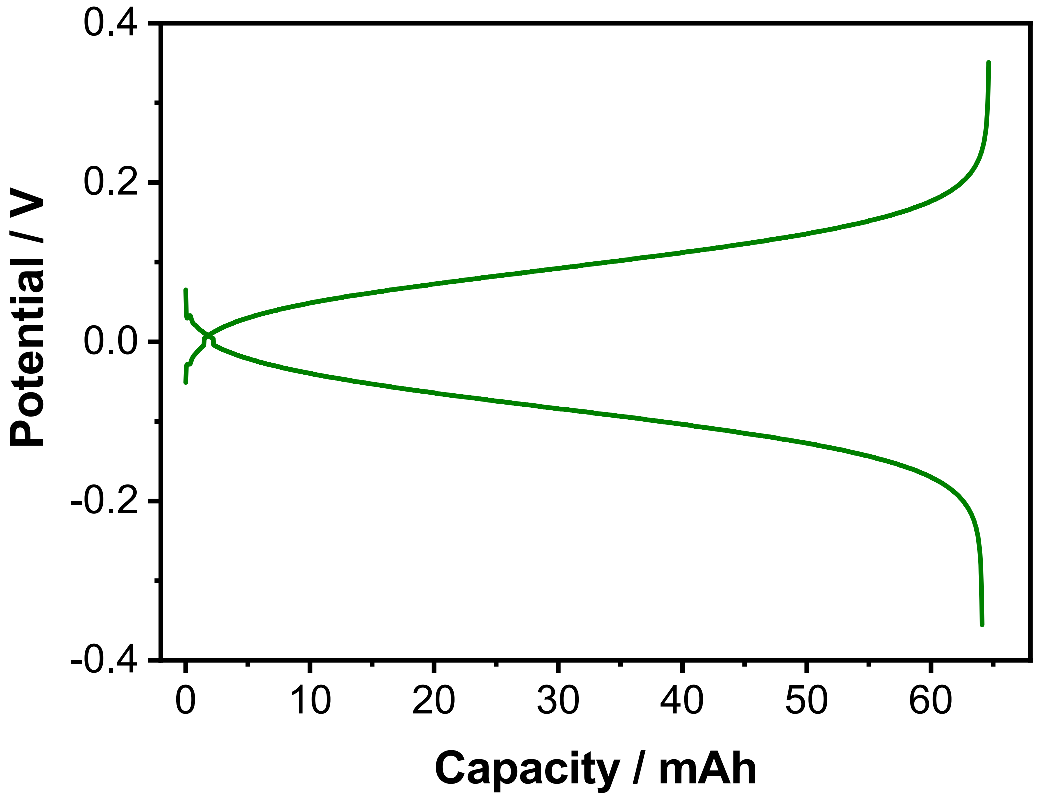

Figure S4. Voltage profile during oxidation and reduction (first cycle) in a symmetric cell with a CLS of 12 mL 0.2 M $K_4Fe(CN)_6$ and a NCLS of 50 mL of a mixture of 0.2 M $K_3Fe(CN)_6$ and 0.1M $K_4Fe(CN)_6$. The current density applied was 10 mA cm^{-2} with voltage cut-offs of ± 0.35V. The oxidation capacity in the first cycle was 64.6 mAh, and the reduction capacity was 64.1 mAh, corresponding to a first cycle efficiency of 99.2 %.

Section S4. Reduction capability of LFP

	Capacity / mAh	
Step	With pellets	Without pellets
Oxidation	81.3	81.1
Reduction	77.6	77.5
Oxidation	77.7	77.5
Reduction	77.8	77.6
Oxidation	77.8	77.6
OCP	0.0	0.0
Oxidation	41.6	0.1
OCP	0.0	0.0
Oxidation	18.5	0.1
Reduction	78.6	77.9
Oxidation	78.7	77.8

Table S1. Capacity values of the oxidation and reduction steps of two electrochemical flow cells with 15 mL of 0.2 M $K_4Fe(CN)_6$ as the CLS solution and 100 mL of 0.1 M $K_3Fe(CN)_6$ and 0.1 M $K_4Fe(CN)_6$ as the NCLS solution at a constant current density of 20 mA/cm², with voltage cut-offs set at 0.35V and -0.35V. LFP pellets were introduced only in one of these cells at the beginning of the first OCP period. The additional charge provided after the OCP highlighted in blue confirm the occurrence of the spontaneous charge transfer between LFP and $K_3Fe(CN)_6$

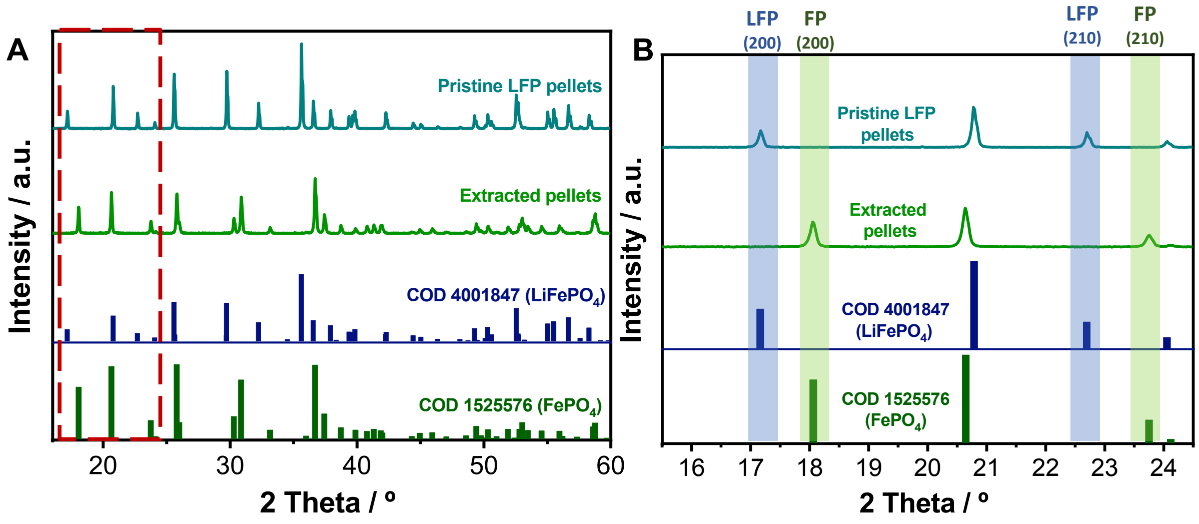

Figure S5. Full (A) and zoomed (B) XRD patterns of the pristine LFP pellets and oxidized pellets extracted from the CLS compartment of the $K_4Fe(CN)_6$ /$K_3Fe(CN)_6$ electrochemical flow cell.

Section S5. Efficiency of the rebalancing process with different amounts of LFP and long-term performance

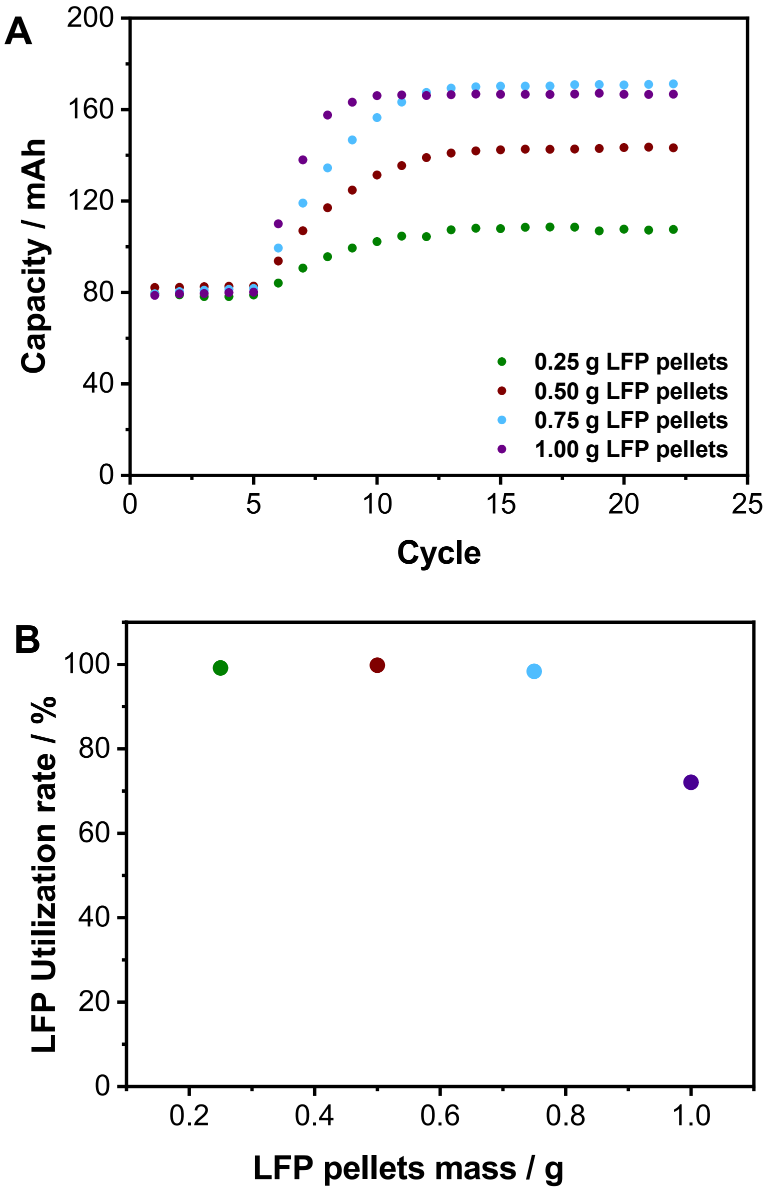

Figure S6. (A) Evolution of charge capacity by adding different amounts of LFP pellets during oxidation/reduction cycling (applying a galvanostatic protocol with a current density of ±20 mA cm^{-2} and ±0.35V cut-off voltage) of a symmetric flow cell with 50

mL of 0.2 M $K_3Fe(CN)_6$ in the NCLS and 22.5 mL of 0.15 M $K_4Fe(CN)_6$ and 0.15 M $K_3Fe(CN)_6$ in the CLS. (B) Utilization rate of the LFP pellets (cycle 11) for the different amounts of LFP pellets added. The utilization rate was calculated as the ratio between the increase in charge storage capacity after adding the pellets and the theoretical charge capacity of the added LFP.

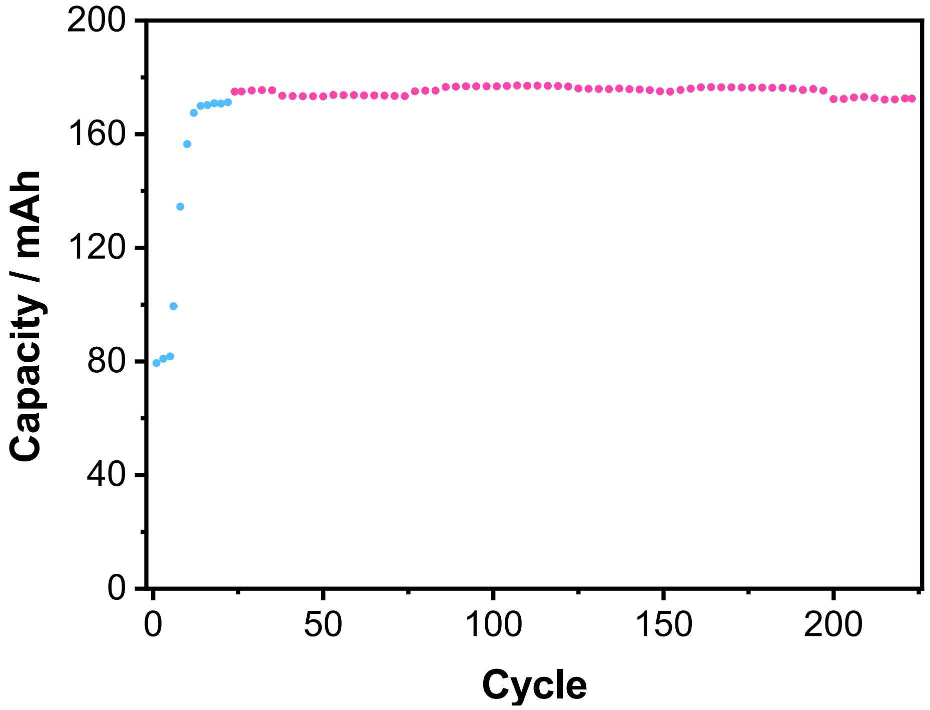

Figure S7. Evolution of charge capacity during oxidation/reduction cycling of a $K_3Fe(CN)_6/K_4Fe(CN)_6$ symmetric cell rebalanced with 0.75 g of LFP pellets. Volume and composition of CLS and NCLS are given in caption of Figure S6. The symmetric cell was cycled applying i) for the first 20 cycles a galvanostatic protocol at ±20 mA cm^{-2} with voltage cut-off of with voltage of ±0.35 V, and ii) the last 200 cycles a potentiostatic protocol with voltage of ±0.35 V and current density cut-offs of ±10 mA cm^{-2} (over a week).

Section S6. Irreversible oxidation of LFP in $K_4Fe(CN)_6$ catholyte

Step	Capacity / mAh
Oxidation	80.1
Reduction	78.8
Oxidation	78.9
Reduction	78.9
OCP	0.0
Reduction	0.2
OCP	0.0
Reduction	0.1
Oxidation	79.3
Reduction	79.0

Table S2. Capacity values of the oxidation and reduction steps of an electrochemical flow cell with 15 mL of 0.2 M $K_4Fe(CN)_6$ as the CLS solution and 100 mL of 0.1 M $K_3Fe(CN)_6$ and 0.1 M $K_4Fe(CN)_6$ as the NCLS solution at a constant current density of 20 mA/cm², with voltage cut-offs set at 0.35V and -0.35V. FP pellets were introduced in the CLS at the beginning of the first OCP period. The absence of additional charge after the OCP highlighted in blue confirms that the charge transfer between LFP and $K_3Fe(CN)_6$ is irreversible (FP does not react with $K_4Fe(CN)_6$).

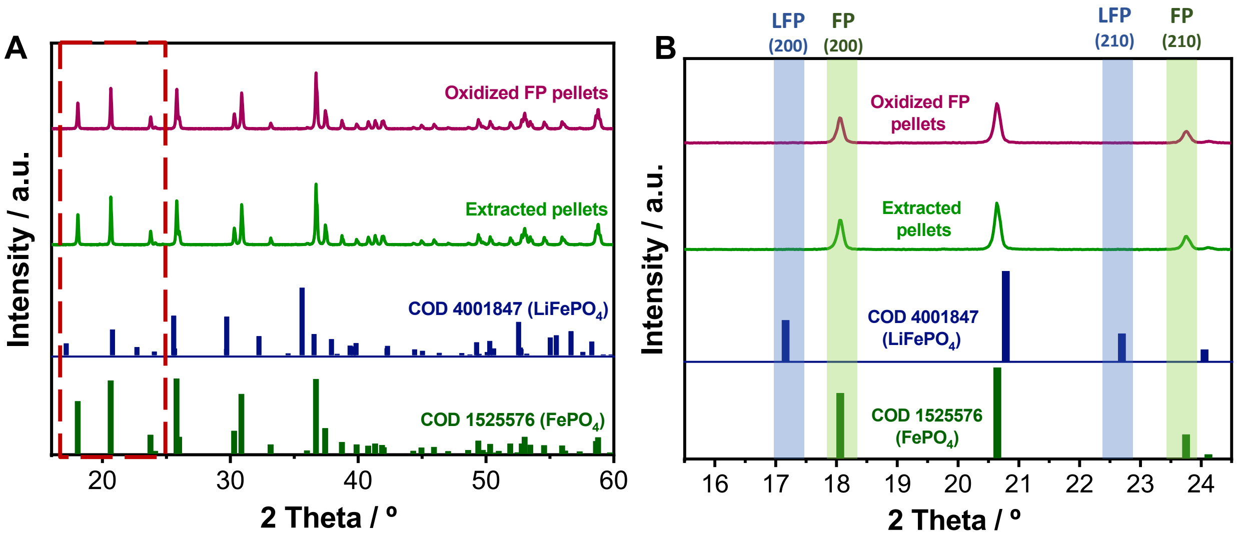

Figure S8. Full (A) and zoomed (B) XRD patterns of the FP pellets and FP pellets extracted from the CLS compartment of the $K_4Fe(CN)_6$/$K_3Fe(CN)_6$ electrochemical flow cell.

Section S7. UV-Vis calibration for determination of $K_3Fe(CN)_6$ concentration

Ultraviolet-visible (UV-Vis) absorption spectroscopy was used to determine the concentration of $K_3Fe(CN)_6$ within the battery tank. The calibration was conducted over a concentration range of 0 mM to 0.6 mM of $K_3Fe(CN)_6$, employing 1 M KCl as supporting electrolyte. A total of seven solutions were prepared, and for each solution, an absorbance spectrum from 200 to 600 nm was recorded. A quartz cuvette with a 1 cm optical path length was chosen for its transparency in the UV range. A calibration curve was subsequently constructed using absorbance measurements at a wavelength of 420 nm (Figure S6).

The absorbance measured from dilution of a 5 μL aliquot of the catholyte in 2 mL was 0.148 a.u. Utilizing the calibration curve and accounting for the dilution factor, the concentration of $K_3Fe(CN)_6$ in the catholyte was determined to be 56.7 mM.

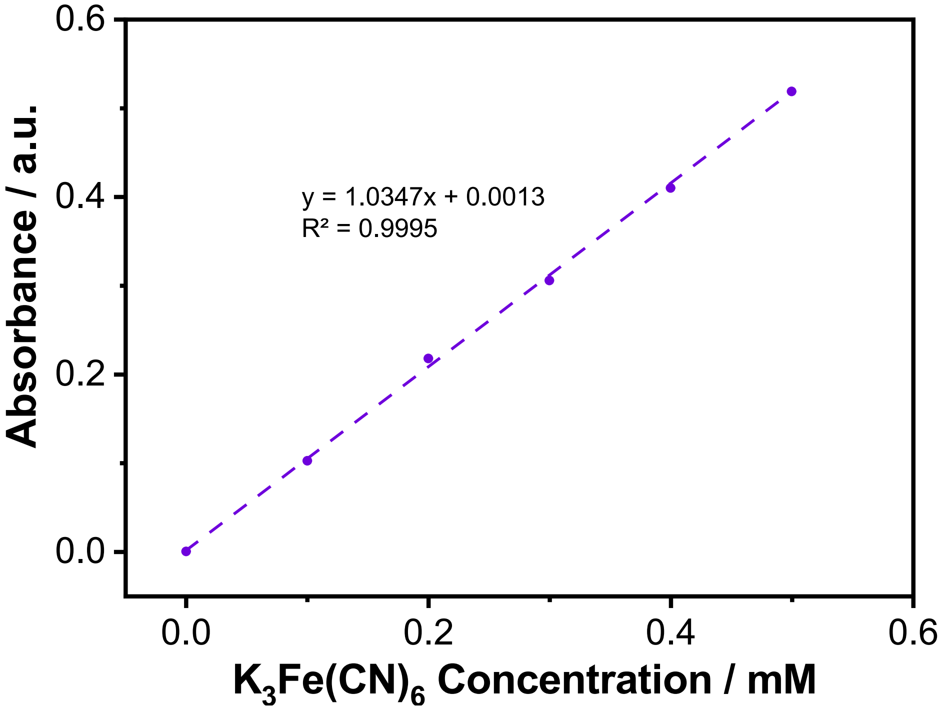

Figure S9. Calibration curve of absorbance at 420 nm vs. concentration of $K_3Fe(CN)_6$.

References

(1) Rubio-Presa, R.; Lubián, L.; Borlaf, M.; Ventosa, E.; Sanz, R. Addressing Practical Use of Viologen-Derivatives in Redox Flow Batteries through Molecular Engineering. *ACS Mater Lett* **2023**, *5* (3), 798–802. https://doi.org/10.1021/acsmaterialslett.2c01105
